# The psychological impact of fertility treatment suspensions during the COVID-19 pandemic

**DOI:** 10.1371/journal.pone.0239253

**Published:** 2020-09-18

**Authors:** Jennifer L. Gordon, Ashley A. Balsom

**Affiliations:** Department of Psychology, University of Regina, Regina, Saskatchewan, Canada; Medical University of Vienna, AUSTRIA

## Abstract

**Purpose:**

To examine the psychological impact of fertility treatment suspensions resulting from the COVID-19 pandemic and to clarify psychosocial predictors of better or worse mental health.

**Methods:**

92 women from Canada and the United States (ages 20–45 years) whose fertility treatments had been cancelled were recruited via social media. Participants completed a battery of questionnaires assessing depressive symptoms, perceived mental health impact, and change in quality of life related to treatment suspensions. Potential predictors of psychological outcomes were also examined, including several personality traits, aspects of social support, illness cognitions, and coping strategies.

**Results:**

52% of respondents endorsed clinical levels of depressive symptoms. On a 7-point scale, participants endorsed a significant decline in overall quality of life (M(SD) = -1.3(1.3), p < .0001) as well as a significant decline in mental health related to treatment suspensions on a scale from -5 to +5 (M(SD) = -2.1(2.1), p < .001). Several psychosocial variables were found to positively influence these outcomes: lower levels of defensive pessimism (r = -.25, p < .05), greater infertility acceptance (r = .51, p < .0001), better quality social support (r = .31, p < .01), more social support seeking (r = .35, p < .001) and less avoidance of infertility reminders (r = -.23, p = .029).

**Conclusion:**

Fertility treatment suspensions have had a considerable negative impact on women’s mental health and quality of life. However, these findings point to several protective psychosocial factors that can be fostered in the future to help women cope.

## Introduction

One in six reproductive-aged couples experience infertility, defined as being unable to achieve pregnancy despite ≥12 months of focused attempts to conceive [1]. Although male and female-factor infertility are equally prevalent, women generally bear the brunt of infertility-related burden: even in cases of male-factor infertility, treatments such as intrauterine insemination (IUI) or in vitro fertilization (IVF) require that women attend near-daily ultrasounds, self-inject gonadotropins, and undergo invasive and painful procedures. Women also carry a disproportionate share of the psychological burden associated with infertility, with infertile women consistently reporting lower self-esteem, more depression and anxiety, and lower life satisfaction, than their male partners [2]. Studies of women presenting for the evaluation of infertility in a tertiary care setting suggest that approximately 30–40% of these women experience clinically significant depression or anxiety [3–6]. In fact, quality of life and levels of depression and anxiety among women undergoing fertility treatments are indistinguishable from those of individuals undergoing cancer treatments and cardiac rehab following a heart attack [7].

On March 17^th^, 2020, the American Society of Reproductive Medicine and the Canadian Fertility and Andrology Society announced their recommendations to immediately suspend all in-person fertility treatments throughout Canada and the U.S. indefinitely due to the COVID-19 pandemic [8, 9]. These recommendations included delaying the start of new treatment cycles but also, in many cases, abandoning treatment cycles that had already begun. While perhaps the right decision given the circumstances, this was devastating news for thousands of couples: unsurprisingly, one early survey of patients at a New York City fertility clinic found that 55% of patients reported being “very” to “extremely” upset over the cancellation of fertility cycles [10]. However, to our knowledge, there have been no studies examining the mental health impacts of fertility treatment suspensions. This was therefore the purpose of the current investigation. Furthermore, the current study aimed to clarify predictors of better or worse mental health during this challenging time.

## Methods

### Participants

Women from across Canada and the United States were recruited to participate in the current online study via advertising on social media. To qualify, women had to report having had their fertility treatments suspended due to the COVID-19 pandemic and that their treatments had not yet resumed. Individuals were compensated with a $15.00 Amazon e-gift card for their participation. The consent form for this study consisted of the first page of the survey—all participants provided consent by clicking on the “Accept” button and continuing to the survey itself. The study was reviewed and approved by the University of Regina Research Ethics Board.

The study was advertised on Facebook between April 26^th^ and June 13^th^, 2020. The study ad instructed prospective participants to message the research team if they were interested. A member of the team then verified their eligibility to participate. If deemed eligible, they were provided with a link and password to access an online survey using Qualtrics survey software.

### Measures

#### Demographic and reproductive history questionnaire

Demographic characteristics such as age, ethnicity, education level, marital status, and household income were obtained. Details about reproductive history were also obtained and participants were asked to identify any diagnoses known to contribute to their infertility and to report which treatments had been suspended due to the pandemic.

#### Patient Health Questionnaire-9 (PHQ-9)

The PHQ-9 [11] consists of 9 items based on DSM-IV criteria for diagnosing depressive disorders and is capable of determining both disorder presence and severity. Items are scored on a 4-point Likert scale ranging from 0 (*Not at all*) to 3 (*Nearly every day*), which indicates the degree participants have been bothered by the listed problems in the past 2 weeks (total scores ranging from 0–27). In the current study, internal consistency was found to be α = .84. An internal consistency above 0.7 suggests that the items of a scale are sufficiently correlated as to suggest that they measure the same general construct.

#### Intolerance of Uncertainty Scale short form-12 (IUS-12)

The IUS-12 [12] includes 12 items on a 5-point scale ranging from 1 (*Not at all characteristic of me*) to 5 (*Entirely characteristic of me*). The questionnaire features three subscales: inhibitory anxiety, prospective anxiety, and a total score. In the current study, internal consistency was found to be α = .87.

#### Revised Life Orientation Test (LOT-R)

The LOT-R [13] is a 10-item questionnaire focusing on pessimism and optimism. The LOT-R is on a 5-point scale ranging from 0 (*strongly disagree*) to 4 (*strongly agree*). Of the 10-items, 6 items contribute to an overall optimism score and 4 are filler questions. In the current study, internal consistency was found to be α = .80.

#### Revised Defensive Pessimism Questionnaire (DPQ-R)

The DPQ-R [14] is a 17-item questionnaire focusing on defensive pessimism. The DPQ-R is on a 7-point Likert scale for a maximum total score of 119. In the current study, internal consistency was found to be α = .71.

#### Illness Cognition Questionnaire (ICQ)

The ICQ [15] is an 18-item questionnaire with responses on a 4-point scale from “Not at all” to “Completely”. For the purposes of this study, the words “my illness” were replaced with “my infertility. This measure has a three-factor structure, these factors being “helplessness” (e.g. my infertility controls my life”), “acceptance” (e.g. “I have learned to live with my infertility”), and “perceived benefits” (e.g. “I have learned a great deal from my infertility”.

#### Infertility coping questionnaire

This questionnaire (S1 File) was developed by our research team because a comprehensive infertility-specific coping questionnaire was not deemed to exist. Its creation was based on a careful review of all existing infertility-specific coping questionnaires. It began as a list of 117 items, which was reduced to 39 items after redundant items were removed, in collaboration with our patient-advisors. These 39 items were retained to represent each of the following subscales: 1) avoidance, 2) active coping, 3) find meaning, 4) defensive pessimism, 5) optimism, 6) seek social support, 5) behavioral engagement. Responses are on a 5-point scale from “Not at all” to “Always”. Its internal consistency in the current study was found to be α = .81.

#### Indicators of social support

Participants were asked to indicate the number of people, apart from their romantic partner, with whom they speak openly about their infertility. They were also asked to rate, on a 5-point scale from ‘Very unhelpful’ to ‘Very helpful’, the degree to which their partner and the degree to which others have helped them cope with the suspension of fertility treatments.

#### Psychosocial impact of treatment suspensions

Participants were asked to rate on a scale from -5 (very negative impact) to +5 (very positive impact), the extent to which their mental health had been impacted by treatment suspensions. They were also asked to rate the extent to which their mental health had been impacted by the other components of the pandemic (e.g. worries about the virus, living with restrictions on daily activities, unemployment, financial difficulties).

#### Quality of life before and after the pandemic

A 1-item quality of life measure [16] asked participants to answer the following question: “Taking everything in your life into account, please rate your overall quality of life on the following 7-point scale. One (1) means life is very distressing; it’s hard to imagine how it could get much worse. Seven (7) means life is great; it’s hard to imagine how it could get much better. Four (4) means life is so-so, neither good nor bad.” They were asked to answer this question twice: first, with the present in mind and second, thinking about their state in the month leading up to treatment suspensions.

### Data analysis

All analyses were conducted using SAS 9.4. The two primary outcomes for this study were change in quality of life rating (from before treatment suspensions) and mental health impact ratings. The Wilcoxon Signed Rank test, a non-parametric test that does not require data to be normally distributed, was used to assess whether change in quality of life and mental health impact were significantly different from 0. Spearman correlations, also a non-parametric test that does not require normally-distributed data, were used to examine the relationship between indicators of social support, personality traits, aspects of illness cognition, and coping strategies, on these two outcomes. For both illness cognitions and coping strategies, an additional linear regression analysis using PROC GLM was conducted including all cognitions or coping factors as predictors within the same model to clarify their independent effects. These were repeated after log-transforming both change in quality of life rating and mental health impact ratings to improve normality of these dependent variables, both of which were left-skewed (towards the negative end of the scale).

### Power analyses

Power calculations were carried out in G*Power. Setting alpha at 0.05, this study had 80% power to detect a correlation coefficient of 0.3, which we judged to be the smallest clinically significant effect that we would want to be powered to detect.

## Results

### Baseline characteristics

A total of 187 women contacted us through Facebook with interest in the study. Thirty-eight participants did not pursue the conversation past an initial explanation of the study and 43 were deemed ineligible to participate. Finally, 14 participants were removed from all analyses because the survey took less than 5 minutes to complete, raising questions about the validity of responses. Thus, 92 participants were remaining for the current analysis.

Table 1 reports the demographic and reproductive health characteristics of these 92 women. Participant ages ranged from 20 to 45 years and time spent trying to conceive ranged from 5 to 180 months. More than half of the participants had had an IVF cycle cancelled and approximately one third were in the midst of IUI. 36% of the participants were ages 35–39 and 12% were aged 40 or older.

**Table 1:** Sample characteristics.

	Mean (SD) or %
**Demographic Characteristics**	
Mean age (SD)	34.2 (4.6)
Country of residence	
Canada	54%
United States	46%
Race/ethnicity	–
White/Caucasian	74%
Black/African-American	3%
Hispanic/Latina	8%
Other	14%
% University degree	66%
Mean annual household income	$70,000 to $89,999
**Reproductive Health Characteristics**	
Months trying to conceive (SD)	36.0 (33.0)
% Nulliparous	59%
Causes of Infertility	–
Male-factor infertility	22%
Problems with egg quality/quantity	10%
Polycystic ovarian syndrome	26%
Endometriosis	8%
Fallopian tube blockage	8%
Unexplained	21%
Other	9%
Suspended Intervention	–
In vitro fertilization	54%
Intrauterine insemination	35%
Frozen embryo transfer	5%
Other	6%
**Mental Health Indicators**	
Patient History Questionnaire-9 Score (/27; 10 = clinical cut-off)	10.0 (5.4)
% with PHQ-9 score ≥10	52%
Median quality of life before (/7)	5.0 (1.1)
Median quality of life now (/7)	4.0 (1.4)
Median mental health impact of treatment suspensions (-5 to +5)	-3.0 (2.1)
Median mental health impact of other pandemic components (-5 to +5)	-1.9 (1.9)
**Social Support Indicators**	
Mean # of confidants about infertility	2.6 (3.0)
0	18%
1–2	48%
3–4	21%
5+	13%
Partner helpfulness rating (1–5)	3.5 (1.2)
1–2 (Very or somewhat unhelpful)	20%
3 (Neither helpful nor unhelpful)	22%
4–5 (Very or somewhat helpful)	58%
Others helpfulness rating (1–5)	3.1 (1.2)
1–2 (Very or somewhat unhelpful)	35%
3 (Neither helpful nor unhelpful)	26%
4–5 (Very or somewhat helpful)	42%
**Personality Traits**	
Defensive Pessimism (/119)	54.9 (8.6)
Optimism (14–18 = moderate; 19+ = high)	18.4 (4.4)
Intolerance of Uncertainty (/60)	35.0 (8.4)
**Illness Cognition Factors, mean item endorsement (1 = not at all; 4 = completely)**	
Helplessness	3.5 (1.1)
Acceptance	3.2 (0.8)
Benefit-finding	3.5 (1.0)

### The psychological impact of fertility treatment suspensions

**Fig 1:**
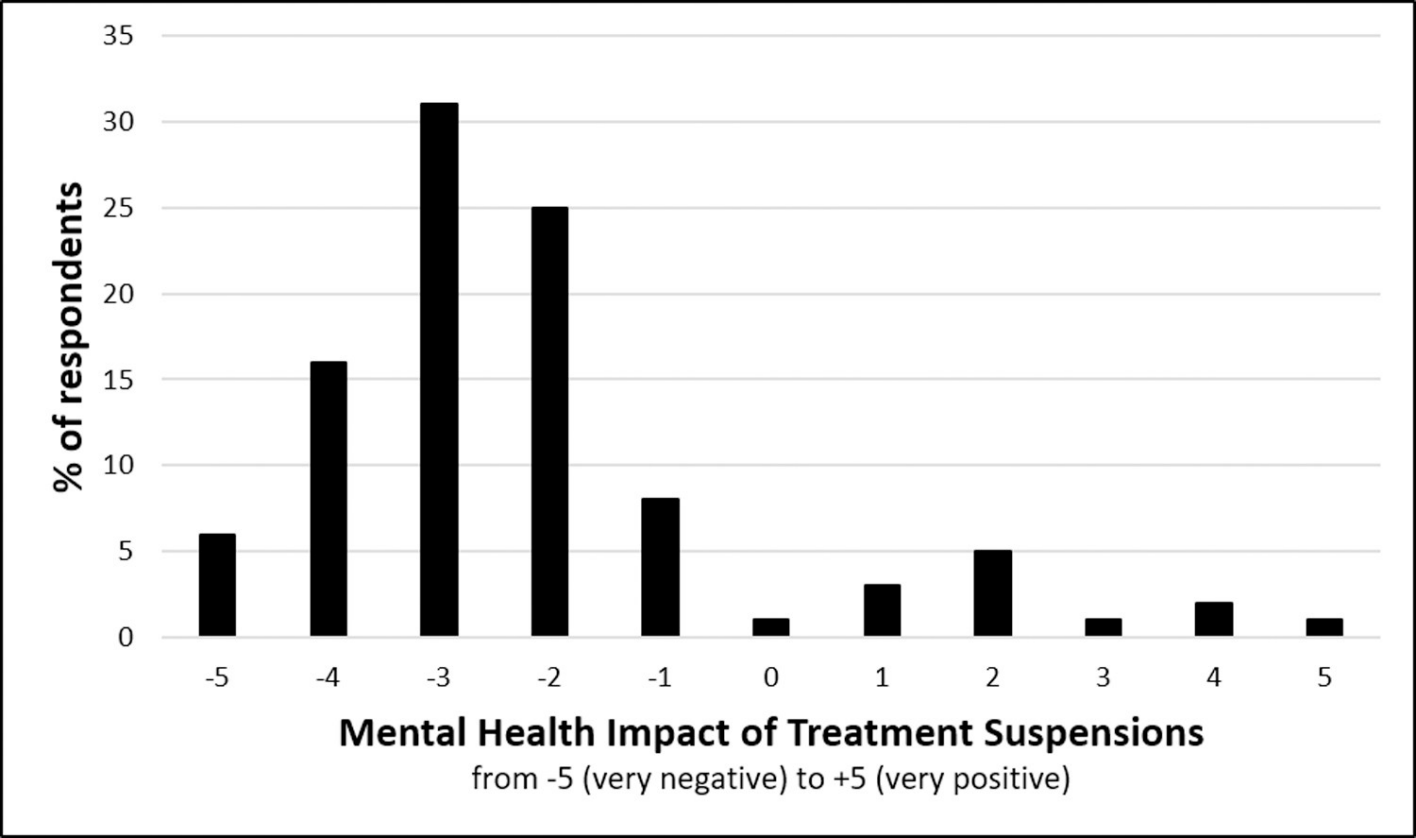
Perceived mental health impact of fertility treatment suspensions.

Table 1 summarizes the median psychosocial variables assessed in the study. Median depressive symptoms were quite high based on the Patient History Questionnaire, with over half of the sample scoring above the clinical cut-off of 10. There was a statistically significant decline in self-reported quality of life of 1.3/7 following the suspension of fertility treatments (S(88) = -1221, p < .0001). When rating the mental health impact of treatment suspensions on a scale from -5 (very negative impact) to +5 (very positive impact), respondents endorsed a median score of -3.0, which is significantly different from 0 (S(86) = -1481, p < .0001) (Fig 1). Overall, 86% of respondents reported that treatment suspensions had had a negative impact on mental health.

### Predictors of the psychological impact of treatment suspensions

#### Baseline characteristics

Neither age, years of education, annual income, nor the number of children a woman had were correlated with either change in quality of life or perceived mental health impact (p >.05). However, the length of time a woman had been trying to conceive was associated with a greater negative perceived mental health impact (r(77) = -.30, p = .008) of treatment suspensions.

#### Personality traits

Table 2 depicts the correlation between three personality traits (trait optimism, defensive pessimism, and intolerance of uncertainty) that were considered potentially relevant under the current circumstances, in relation to the overall change in quality of life and the mental health impact attributed to fertility treatment suspensions. With regards to personality traits, defensive pessimism was related to a greater negative change in overall quality of life as well as a greater negative mental health impact of treatment suspensions. Neither trait optimism nor intolerance of uncertainty were significantly related to any outcomes (p >.05).

**Table 2:** Spearman correlations between personality traits and infertility-related cognitions with mental health outcomes.

	Δ Quality of Life	Mental Health Impact
**Personality Traits**		
Defensive pessimism	-.25*	-.31**
Optimism	.15	.21
Intolerance of Uncertainty	.07	-.15
**Social Support Indicators**		
# of confidants	.10	.16
Partner helpfulness rating	.00	.28**
Others helpfulness rating	.17	.31**
**Infertility-related Cognitions**		
Acceptance	.32**	.50***
Helplessness	-.02	-.33**
Benefit-Finding	.17	.36**
**Coping Strategies**		
Avoidance	.00	-.23*
Active coping	.02	-.06
Give meaning	.13	.13
Pessimism	.07	.01
Optimism	.04	.17
Seek social support	.35***	.11
Behavioral engagement	.16	.10

*p < .*05*,

***p < .*001*.

#### Infertility-related cognitions

**Fig 2:**
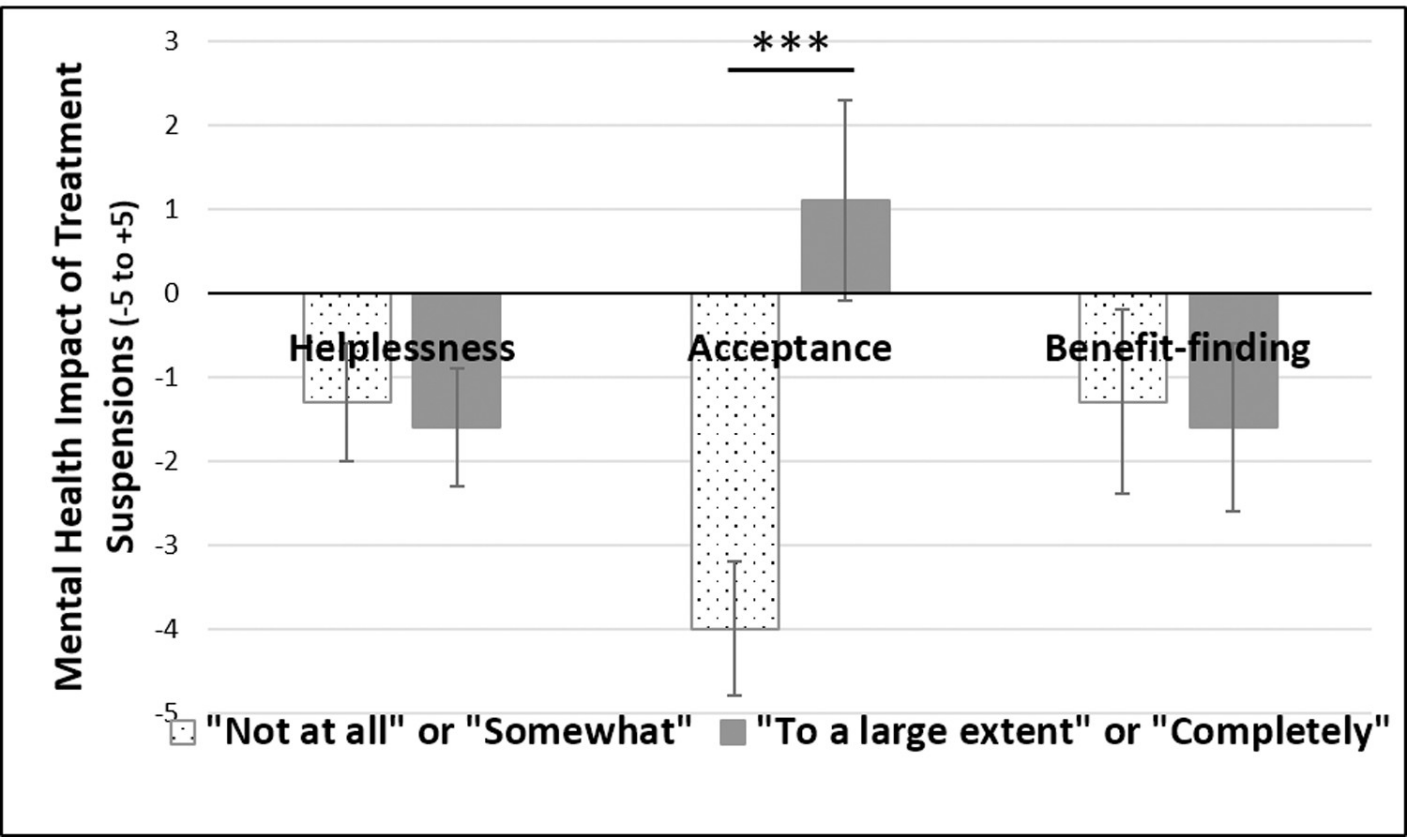
Moderation of mental health impact of fertility treatment suspensions by infertility-related cognitions. ***p < .0001.

Greater acceptance was associated with a more positive change in overall quality of life and lesser mental health impact attributed to treatment suspensions (Table 2). Furthermore, greater benefit-finding was associated with a smaller mental health impact of treatment suspensions while helplessness was associated with a greater negative mental health impact of treatment suspensions. However, when all three illness cognition factors were included in the same regression model as three simultaneous predictors of quality of life change, only acceptance was a significant predictor of treatment suspensions (β(SE) = 0.8(0.3), p < .01) while helplessness (β(SE) = 0.3(0.2), p = .163) and benefit-finding (β(SE) = 0.1(0.2), p = .831) were not. The same pattern was seen when examining the impact of illness cognitions on perceived mental health impact: acceptance was a significant predictor (β(SE) = 0.3(0.1), p < .001) while helplessness (β(SE) = 0.0(0.1), p = .592) and benefit-finding (β(SE) = 0.1(0.1), p = .229) were not. These results remained unchanged when quality of life change and mental health impact were log-transformed to improve normality of the dependent variable. Fig 2 depicts the mean perceived impact by mean response to the Illness Cognition Questionnaire.

#### Coping strategies

The internal consistency of each of the seven subscales was examined. In instances where it was below 0.7, items were removed to improve it. This resulted in 7 items being removed. The remaining 32 items are included in Table 3. The internal consistency of the final subscales are as follows: 1) avoidance, α = 0.82, 2) active coping, α = 0.71, 3) finding meaning, α = 0.72, 4) defensive pessimism, α = 0.66, 5) optimism, α = 0.80, 6) seek social support, α = 0.77, and 7) behavioural engagement, α = 0.67.

**Table 3:** Endorsement of strategies for coping with the suspension of fertility treatments.

	Endorsement (% “Often” or “Always”
**Avoidance/ Denial**	
Avoid reminders of my difficulty conceiving	40
Hide my feelings about getting pregnant from others	59
Tell myself to stop when I start thinking about trying to get pregnant	49
Fill my downtime to avoid thinking about getting pregnant	42
Avoid thinking about the future	40
Avoiding social situations	25
Pretend that my trouble getting pregnant does not bother me	32
Use food, non-prescription drugs, or alcohol to help myself cope	51
**Active Coping**	
Act towards finding a solution to my difficulties conceiving	48
Focus on exploring next steps to pursue if my current attempts fail	44
Seek information or advice that can help me achieve pregnancy	40
**Finding Meaning**	
Take time to understand, identify, or express my feelings	19
Try to find meaning in my experience	26
Try to grow as a person as a result of this experience	28
Accept the situation as it is	16
**Defensive Pessimism**	
Decide that I don’t care	8
Try to keep my expectations low	50
Prepare myself for the worst	42
Tell myself that it would be for the best if I didn’t get pregnant	17
Tell myself that having biological children is not important	29
**Optimism**	
Believe that everything will work out	33
Stay optimistic that my efforts will be successful	33
Fantasize about how things might turn out	55
Believe that I will feel better in time	32
Try to find humor where I can	32
**Seek social support**	
Seek emotional support professionals (e.g., counsellor, doctor)	15
Seek emotional support from friends or loved ones	22
Seek emotional support on the internet (e.g., blogs, chatrooms)	33
Seek emotional support from others with similar experience	24
**Behavioral Engagement**	
Practice self-care (e.g., meditation, watch movie)	26
Focus on caring for others (e.g., loved ones, volunteering)	34
Focus on other life goals (e.g., take a new class, focus on my career)	32

As shown in Table 2, Spearman correlations revealed that seeking social support was correlated with a more favourable change in quality of life and that engaging in less avoidance was associated with a more favourable change in mental health. When all seven coping factors were included in the same regression model, seeking social support remained the only factor predicting change in quality of life: specifically, a 1-point increase in mean item endorsement was associated with a 0.6-point increase in quality of life (β(SE) = 0.6(0.2), p = .004). Avoidance remained predictive of a greater negative mental health impact (*β(SEM)* = -0.7(3), *p* = .033). However, optimism emerged as predictive of a more favourable mental health impact of treatment suspensions (*β(SEM)* = 0.8(0.3), *p* = .015). The other coping factors were not significant predictors (p > .05). These results remained unchanged when quality of life change and mental health impact were log-transformed.

#### Sensitivity analyses: Predictors of the mental health impact of other pandemic aspects

On a scale from -5 to +5, participants were asked to reflect on the extent to which their mental health had been impacted by aspects of the COVID-19 pandemic that were unrelated to their fertility treatments being cancelled (e.g. activity restrictions, social isolation, financial hardship, job loss). Of the variables listed in Table 2, defensive pessimism (r(83) = -.29, p = .007), infertility acceptance (r(83) = .24, p = .024), and infertility benefit-finding (r(83) = .22, p = .049) were weakly correlated with this outcome.

## Discussion

The current study sought to examine the mental health impact of fertility treatment suspensions resulting from the COVID-19 pandemic. Furthermore, it sought to explore the psychosocial predictors of this psychological impact. Overall, results suggest that the mental health impact of treatment suspensions is substantial: indeed, over 50% of respondents reported clinically significant depressive symptoms, a rate that is considerably higher than typical rates observed in this population, which hover closer to 30% (Nelson, Shindel, Naughton, Ohebshalom, & Mulhall, 2008; Volgsten et al., 2008). Similarly, over 50% of respondents endorsed a “-3” or worse on a scale from -5 to +5 to reflect the mental health impact that treatment suspensions have had. These findings mirror a survey of patients at a New York City fertility clinic finding that 55% reported being “very” to “extremely” upset over treatment cancellations [10]. However, the current study extends these findings by conducting a more detailed assessment of the mental health impact of treatment suspensions, as well as factors moderating this impact.

Several variables were found to moderate the psychological impact of treatment suspensions. Perhaps unsurprisingly, women who had been trying to conceive for a longer time reported a greater negative mental health effect of treatment suspensions. Furthermore, while the number of confidants a person had was unrelated to the impact of treatment suspensions, women who reported better emotional support from their partner and others also reported a smaller negative mental health impact. This is consistent with prior research finding that better quality social support is associated with lower infertility-related distress [17].

It is noteworthy that the mean number of reported confidants was very small and that only 42% of respondents reported that their confidants were at least “somewhat” helpful in their efforts to cope with fertility treatment suspensions. Women found their partners to be somewhat more helpful, however, as 58% were rated as at least “somewhat” helpful. Furthermore, only 22% of women endorsed seeking social support from friends and loved ones at least “Often”. Overall, these findings suggest that quality social support is beneficial for women who are struggling with the stresses of infertility but that this is not available to most women. This is consistent with research finding that unsupportive social interactions are commonly reported by women struggling with infertility and that these interactions predict greater distress [18, 19]. Thus, interventions aimed at improving the quality of the social support that they receive from others may be beneficial for this population. For example, a recent pilot study [20] found that couples experiencing infertility benefited greatly from 12 sessions of interpersonal therapy, a therapeutic approach that is primarily focused on improving the quality of one’s interpersonal relationships. Furthermore, in this study, interpersonal therapy was found to result in significantly better outcomes when compared to brief supportive therapy.

With regards to personality traits, defensive pessimism was associated with a greater decline in quality of life, as well as a greater negative mental health impact of fertility treatment suspensions. Defensive pessimism is the tendency to, in times of uncertainty, focus on potential negative outcomes and have low expectations in an effort to avoid disappointment [14, 21]. Although this approach may prove useful in motivating an individual to take steps toward avoiding an unwanted outcome under certain circumstances (Spencer & Norem, 1996), our findings suggest that it is maladaptive in the context of the largely uncontrollable condition of infertility.

Spearman correlations revealed that greater acceptance and benefit-finding were associated with a lesser mental health impact of fertility treatment suspensions while greater helplessness was associated with a greater negative impact. However, in a regression model including all three infertility-related cognitions as predictors simultaneously, only acceptance was found to be a significant predictor. The importance of illness acceptance–that is, the extent to which an individual changes their focus from the elimination of their illness to living as well as possible with their illness–has been shown to be a strong predictor of wellbeing among those living with chronic pain [22–24]. Consistent with this, meta-analytic evidence suggests that Acceptance and Commitment Therapy (ACT) [25], which aims to foster greater illness acceptance and reduce illness-related avoidance, is an effective intervention for chronic pain [26]. To our knowledge, only one case study has documented the use of ACT for infertility-related distress [27], with promising effects.

With regards to coping strategies, seeking social support and endorsing greater optimism were associated with better outcomes. In contrast, avoidance of infertility reminders was associated with worse outcomes. This latter finding is consistent with prior research finding that the endorsement of an avoidant coping style has been associated with worse psychological outcomes in the context of infertility [28–31]. Furthermore, in one of the few existing longitudinal studies of coping and psychological outcomes in infertility, the avoidance of reminders and thoughts concerning one’s infertility was found to be a mediator between baseline vulnerability for psychopathology and 12-month distress levels [32]. Interventions targeting avoidance as a coping strategy, such as Acceptance and Commitment Therapy, may be well-suited for infertility-related distress. Although avoidance is often a clinical target in Cognitive Behavioural Therapy, most studies that have applied this treatment approach to infertility have not directly targeted infertility-related avoidance [33, 34].

The current study findings should be interpreted in light of some limitations. First, it is a cross-sectional investigation, providing only a snapshot of the interrelationships between psychosocial factors and infertility-related distress in the context of the COVID-19 pandemic. The sample size was somewhat limited and is also not guaranteed to be representative of all women under similar circumstances–potentially, more distressed women were compelled to complete the survey as it appeared on their Facebook feed. Nonetheless, it provides some valuable insights into the emotional challenges that the COVID-19 pandemic has introduced in the lives of women struggling with infertility. Furthermore, it points to several targets for future interventions specifically targeting infertility-related distress.

In summary, the current study suggests that the suspension of fertility treatments have had a significant negative impact on women’s mental health and quality of life. Low defensive pessimism, high-quality social support, greater infertility acceptance, and less use of avoidance, were all found to be protective factors against the negative effects of treatment suspensions on wellbeing. These findings suggest that additional mental health resources are likely to be needed in this population; they also suggest that infertility-specific psychological interventions should target social support, infertility acceptance, and infertility-related avoidance.

## Supporting information

S1 File The Infertility Coping Questionnaire, developed by the research team for the purposes of this study. (PDF)
