# Supplementary material for: The psychological impact of fertility treatment suspensions during the COVID-19 pandemic
Source: PLoS One. 2020 Sep 18;15(9):e0239253. doi: 10.1371/journal.pone.0239253 (PMC7500693; doi:10.1371/journal.pone.0239253)
Supplement: S1 File — (PDF) [file pone.0239253.s001.pdf]

Infertility Coping Questionnaire

These questions pertain to this cycle or the experience you have right now. Only complete the questions marked with an asterisk (*) if you are currently attempting to conceive.

		Not at all	Rarely	Sometimes	Often	Always
1.	Avoid reminders of my difficulty conceiving (e.g., children, pregnant women, pregnancy magazines, children's TV network)	1	2	3	4	5
2.	Hide my feelings about getting pregnant from others	1	2	3	4	5
3. *	Avoid doing things that increase my likelihood of conceiving (e.g. not tracking my cycle, not following fertility treatments)	1	2	3	4	5
4.	Tell myself to stop when I start thinking about trying to get pregnant	1	2	3	4	5
5.	Fill my downtime with activities to avoid thinking about getting pregnant	1	2	3	4	5
6.	Avoid thinking about the future	1	2	3	4	5
7.	Avoiding social situation (e.g., engaging in isolation, avoiding friends and family)					
8. *	Act towards finding a solution to my difficulties conceiving (e.g., tracking my cycle, pursuing fertility treatments)	1	2	3	4	5
9. *	Focus on exploring next steps to pursue if my current attempts fail.	1	2	3	4	5
10.	Take time to understand, identify, or express my feelings	1	2	3	4	5
11.	Seek information or advice that can help me achieve pregnancy	1	2	3	4	5
12.	Try to find meaning in my experience	1	2	3	4	5
13.	Try to grow as a person as a result of this experience	1	2	3	4	5
14.	Accept the situation as it is	1	2	3	4	5
15.	Tell myself that maybe it would be for the best if I did not achieve pregnancy	1	2	3	4	5

16.	Decide that I do not care about the result	1	2	3	4	5
17.	Prepare myself for the worst	1	2	3	4	5
18.	Tell myself that having biological children is less important to me than I thought	1	2	3	4	5
19.	Think about how I will effectively manage my emotions if I don’t achieve pregnancy this time around	1	2	3	4	5
20.	Try to keep my expectations low when it comes to my chances of achieving pregnancy	1	2	3	4	5
21.	Refuse to believe that I’m having trouble getting pregnant	1	2	3	4	5
22.	Believe that everything will work out<br>(e.g., I will have a child one day, pray for a miracle)	1	2	3	4	5
23.	Stay optimistic that my efforts will be successful	1	2	3	4	5
24.	Pretend that my trouble getting pregnant does not bother me	1	2	3	4	5
25.	Fantasize about how things might turn out	1	2	3	4	5
26.	Believe that I will feel better in time	1	2	3	4	5
27.	Seek spiritual comfort (e.g. attend religious service, speak with clergy)	1	2	3	4	5
28.	Seek emotional support about my problems getting pregnant from professionals (e.g., counsellor, doctor)	1	2	3	4	5
29.	Seek emotional support about my problems getting pregnant from friends or loved ones	1	2	3	4	5
30.	Seek emotional support about my problems getting pregnant on the internet (e.g., blogs, chatrooms)	1	2	3	4	5
31.	Seek emotional support about my problems getting pregnant from others with similar experience (e.g., support group, friend with infertility)	1	2	3	4	5

32.	Focus on my physical health to reduce anxiety levels (e.g., diet, exercise)	1	2	3	4	5
33.	Use strategies to practice self-care (e.g., meditation, watch movie)	1	2	3	4	5
34.	Foster my spirituality or grow my faith (e.g., praying, reading religious text)	1	2	3	4	5
35.	Use food, non-prescription drugs, or alcohol to help myself cope	1	2	3	4	5
36.	Use prescribed medication to help myself cope (e.g., antidepressants, antianxiety medication)	1	2	3	4	5
37.	Try to find humor where I could	1	2	3	4	5
38.	Focus on caring for others (e.g., loved ones, volunteering)	1	2	3	4	5
39.	Focus on other life goals (e.g., take a new class, focus on my career)	1	2	3	4	5
